# Parametric shift from rational to irrational decisions in mice

**DOI:** 10.1038/s41598-020-79949-w

**Published:** 2021-01-12

**Authors:** Nathan A. Schneider, Benjamin Ballintyn, Donald Katz, John Lisman, Hyun-Jae Pi

**Affiliations:** 1 Volen Center for Complex Systems, Neuroscience Program, Department of Biology, Brandeis University, Waltham, MA 02453 USA; 2 Volen Center for Complex Systems, Neuroscience Program, Department of Psychology, Brandeis University, Waltham, MA 02453 USA

**Keywords:** Neuroscience, Psychology

## Abstract

In the classical view of economic choices, subjects make rational decisions evaluating the costs and benefits of options in order to maximize their overall income. Nonetheless, subjects often fail to reach optimal outcomes. The overt value of an option drives the direction of decisions, but covert factors such as emotion and sensitivity to sunk cost are thought to drive the observed deviations from optimality. Many questions remain to be answered as to (1) which contexts contribute the most to deviation from an optimal solution; and (2) the extent of these effects. In order to tackle these questions, we devised a decision-making task for mice, in which cost and benefit parameters could be independently and flexibly adjusted and for which a tractable optimal solution was known. Comparing mouse behavior with this optimal solution across parameter settings revealed that the factor most strongly contributing to suboptimal performance was the cost parameter. The quantification of sensitivity to sunk cost, a covert factor implicated in our task design, revealed it as another contributor to reduced optimality. In one condition where the large reward option was particularly unattractive and the small reward cost was low, the sensitivity to sunk cost and the cost-led suboptimality almost vanished. In this regime and this regime only, mice could be viewed as close to rational (here, ‘rational’ refers to a state in which an animal makes decisions basing on objective valuation, not covert factors). Taken together, our results suggest that “rationality” is a task-specific construct even in mice.

## Introduction

The classical models of economic decision-making, particularly as applied to foraging behavior, present subjects as rational agents who deliberately analyze the cost and benefit associated with available options and optimize their choices in order to maximize gain given the amount of time or quantity of resources available^1–6^. Although this limited definition of optimal behavior can open a philosophical debate, there is no doubt that the principle of this optimization has provided a theoretical foundation capable of explaining and predicting a plethora of phenomena in decision-making.

Often, however, the outcome of decision-making deviates significantly from the optimal outcome^7–10^. This is because the value assessment of given options is continually influenced by covert factors such as the subject’s fluctuating intrinsic state and a constantly changing external environment^11–13^. While the objective value of the given options is determined by overt factors such as the quantity or the calorie content of food, covert factors are subjective in nature and hard to quantify. The objective value is inevitably a major force driving the direction of the choices, but models incorporate both overt and covert factors to explain frequent deviations from optimality^7,14,15^. Much progress has been made in understanding these factors, but questions still remain as to which contexts most drive the deviations from optimality, as well as to the context-dependence of the deviations’ magnitude.

Although research using non-human laboratory animals has provided vast opportunities for investigating the biological substrates and neural mechanisms underlying decision-making, the non-verbal nature of these subjects makes topics such as suboptimality and irrationality difficult to study. Nonetheless, a series of recent studies by the Redish group applied quantitative behavioral readouts and theoretical frameworks to investigate covert decision factors in laboratory animals^9,14,16,17^. These studies reveal animal behavior resembling the sunk cost fallacy, an irrational thought process wherein a subject tends to base a decision on previous investment choices while ignoring more profitable future outcomes^18,19^.

We sought to further understand the impact of the overt and covert factors on decision-making by extending this approach in a mouse model system. Bringing the above-described work together with several past studies on decision-making in the context of foraging behavior^17,20–26^, we devised an economic decision-making task in which cost and benefit parameters can be flexibly and independently adjusted, and in which a tractable optimal solution is always identifiable. Note that the definition of optimality or rationality in this study has limited meanings that are specific to the scope of this study. Here, optimality is defined and calculated in reference to the expectation of ratios (EoR) model in foraging theory^27^ (see “Methods” and “Results”) and rationality refers to a state in which an animal makes decisions basing on objective valuation, not covert factors. In our behavioral task, on each trial mice make the decision to lever press at a progressive or fixed ratio reward option (PR; FR) with different amounts of water reward associated with each^21^. The shift of ratio preference was quantified by the PR requirement at the indifference points where the values of the two sides became equivalent. We tested whether mice adjusted their decisions and indifference point depending on the cost–benefit parameters and found that their switching decisions did indeed shift accordingly.

By comparing the actual behavioral outcomes with the optimal solutions, a deviation from optimality could be quantified and compared across parameter settings. Doing so, we found that costs had a larger effect than benefits on suboptimal performance, measured as the difference between observed and optimal behavior. Furthermore, mice exhibited behavior similar to that observed in sunk cost studies^9,19^, and indeed, further analysis revealed that sunk cost contributed to the suboptimal outcomes. This contribution was highly parameter-specific, however, in that we identified a regime in the parameter space where the cost parameter and susceptibility to sunk cost fallacy had no significant impact. In our task, therefore, mice can be viewed as rational agents in some regimes, and not in others—a human-like behavior. Thus, our approach provides a platform to investigate both rational and irrational decisions in the same task.

## Results

### A flexible economic choice behavior for mice

In order to quantitatively evaluate the factors that influence economic decision-making, we devised a behavioral paradigm that allowed us to flexibly and independently adjust the costs and benefits of the possible choices. In addition, this task has a tractable optimal solution that provided a metric of how optimal mouse behavior was.

**Figure 1:**
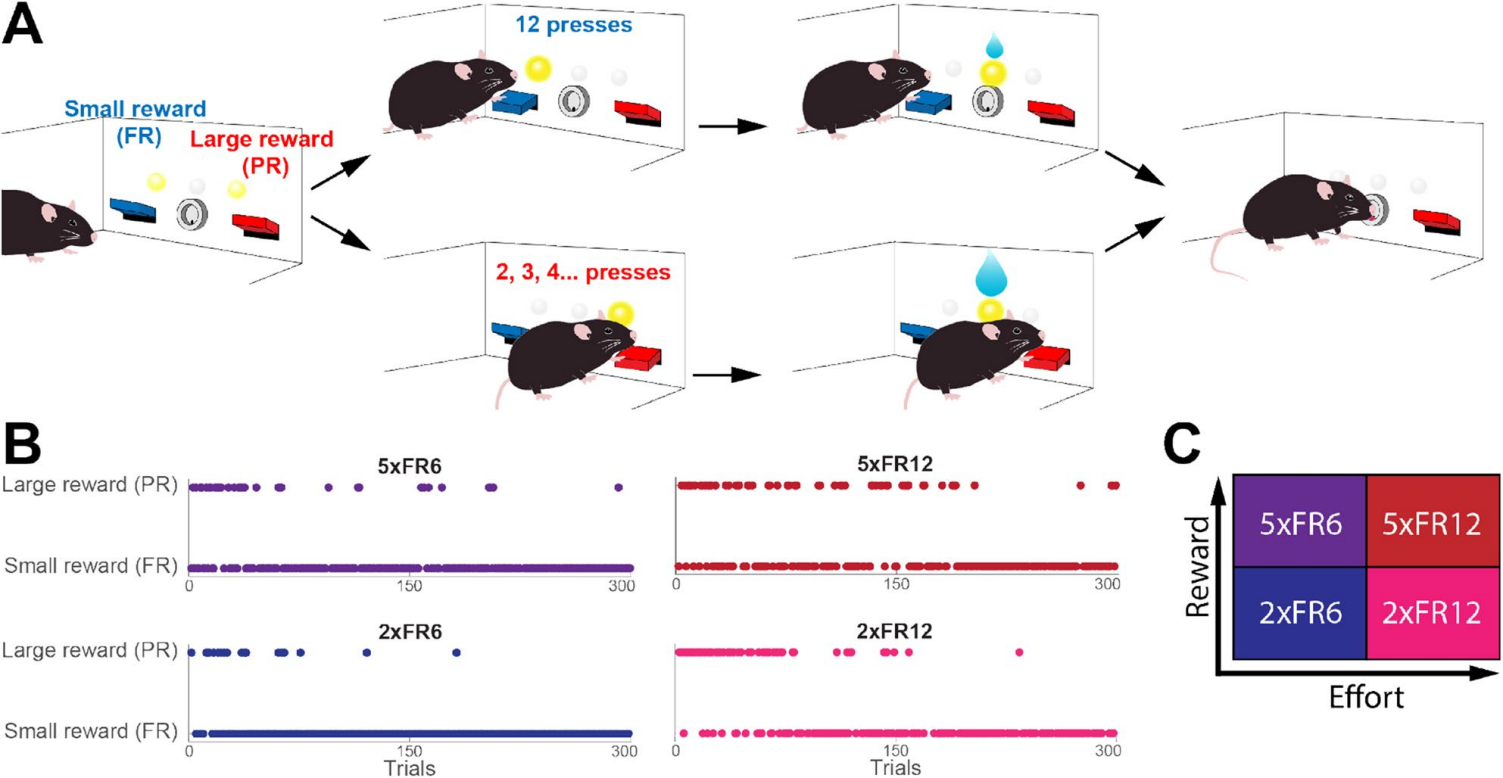
Switching decision task. (**A**) Schematic of task design. (**B**) Example sessions for each parameter pair. Each point is a single trial within the session. Only the first 300 trials of each session are shown. All sessions are from the same animal. (**C**) Grid showing increasing reward and cost for each type of parameter pair. The nomenclature of the parameter pairs shows the ratio of large reward size to small reward size (large reward either ×2 or ×5 larger) as well as the fixed ratio requirement, either 6 presses (FR6) or 12 presses (FR12).

Water-restricted mice were required to press levers to collect a water reward (Fig. 1A). Mice could freely choose to press either a fixed ratio (FR) lever or progressive ratio (PR) lever. The FR lever provided a small volume of water reward each time mice completed a fixed (i.e., unchanging) number of presses (Supplementary Movie S1); the PR lever, meanwhile, provided a large volume of water, but achieving this volume required a number of presses that increased (e.g., 2, 3, 4 …) with each successful reward (Supplementary Movie S2). Mice pre-trained to press both levers reliably preferred the PR lever at the start of each session, undoubtedly because this strategy afforded them a large reward with little effort compared to the FR (Fig. 1B). As the session progressed and the PR requirement became higher, however, this cost (i.e., the required number of presses) overwhelmed the benefit of the large reward, and mice switched their preference to the FR lever, supporting the general hypothesis that mice evaluate the relative values of given choices in terms of both costs and benefits.

By fixing one parameter (e.g., reward amount) and changing the other (e.g., lever press requirement), the contribution of cost and benefit in decision-making can be evaluated semi-independently within the same task, across a wide range of parameter combinations. In this initial study, we chose four combinations of parameters by crossing the ratio of large reward to small reward (2:1 or 5:1) with the number of presses required for the FR reward (6 or 12) and devised a nomenclature to differentiate the parameters. For instance, 2xFR12 means the volume of large reward, 6 µL, is twice as much as that of the small reward, 3 µL, and the FR requirement is 12 presses; the four combinations of parameters can be denoted as 2xFR6, 2xFR12, 5xFR6, and 5xFR12 (Fig. 1C). An equal number of male and female mice were evaluated, but as no significant male–female differences were observed (Supplementary Fig. S1), we combined the data for the below analyses. Four sessions with each parameter combination were collected (160 sessions total: 4 sessions × 4 parameter pairs × 10 mice), and each mouse did one session per day. Left and right levers were pseudo-randomly assigned to be PR and FR at the start of each session. An analysis of the amount of time between lever presses (inter-press interval, IPI) revealed that while there is some variability in IPIs with increasing lever presses, there is not a sizable increase in IPIs until after ~ 60 lever presses (Supplementary Fig. S2), suggesting that this behavioral task does not cause fatigue on later trials.

### Mice adjust switching decisions proportional to the values of session parameters

Having established the basic rationality of task performance, we next asked how the changes in relative reward size (the benefit parameter) and the number of lever presses (the cost parameter) affected the choice behavior. Consistent with our initial observation, all mice were able to make switching decisions by accurately evaluating the cost–benefit relationship of the given choices. The point at which switching decisions were made varied between conditions, however, a fact that is visible in a simple plot of decisions over time (Fig. 1B). The switch to FR happens earliest in the 2xFR6 condition and latest in the 5xFR12 condition, assuredly because of the different relative values of the PR. Using the parameter variables to estimate the relative values of the PR, we see that the value of the large-reward PR is lowest at 2xFR6 because the PR reward is low and the effort and time costs for the FR are also low. Alternatively, the PR value is highest at 5xFR12 because the reward is high and the alternative choice requires more effort and time. The PR values of 2xFR12 and 5xFR6 are somewhere in between the other two. Reflecting these relative values, the number of PR rewards increased as the value increased (Supplementary Fig. S3A). These measures were also quantified in percentage of the PR choices in order to normalize the total number of trials between mice (Supplementary Fig. S3B).

An analysis of the amount of trials that mice failed to complete provided data consistent with the above conclusions. A trial was considered incomplete if the mouse paused activity for more than ten seconds or commenced in pressing the alternative lever after initiating a trial but before reward availability. When the relative values of the PR were higher, mice tried and failed more (Supplementary Fig. S3C,D) and the failure was more frequent on the PR side (Supplementary Fig. S3E, F). Increasing the cost parameter (i.e. number of FR presses) contributed more to the incomplete trials. The cost change also exerted a stronger effect on the task performance as indicated in the total number of trials and water collected (Supplementary Table S1). Consistent with our hypothesis, these results suggest that mice can differentiate the relative values of the session parameters and adjust their decisions accordingly.

### Quantification of switching decisions by indifference points

One idea implemented in our task design was utilizing an “indifference point” as a behavioral readout of how mice evaluate cost and benefit. Theoretically, these points occur when the subjective values of each side are equal. In our behavior task, the PR with large reward is initially more valuable than the FR with small reward; however, as the PR requirement increases, the value of the PR decreases and the FR value remains fixed. At some point, the subjective value of the PR becomes equivalent to that of the FR:

$$ V_{PR} = V_{FR} . $$

**Figure 2:**
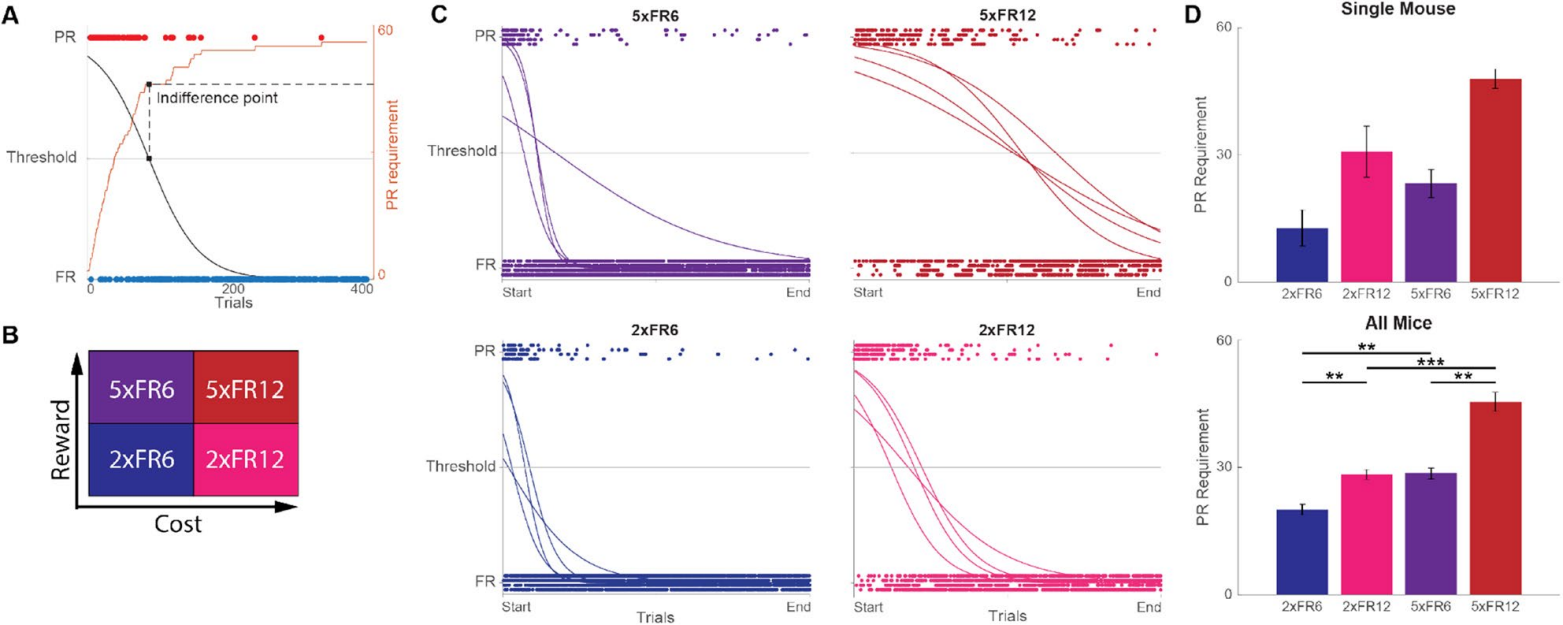
Estimating indifference points through a sigmoid curve shows variation in switching behavior between parameter conditions. (**A**) Example session showing how the indifference point is estimated. Black curve is the sigmoid fit and orange curve is the cumulative PR requirement over time. First, the data is fitted to a sigmoid curve. Then, where the curve crosses the threshold, a trial number is identified (black squares). Finally, the indifference point is determined by finding the number of presses required for the PR at this trial (dashed lines). (**B**) Grid showing increasing reward and cost for each type of parameter pair. (**C**) Sigmoid curves for all 16 sessions of one mouse, sorted by session parameters. Trials are plotted as individual points normalized to the total number of trials in the session and each row of points is a different session. For sessions with higher reward or effort, the threshold intersection shifts farther to the right. (**D**) Indifference points for a single mouse (top, n = 4 sessions) and the entire population (bottom, n = 10 mice). Error bars reflect standard error of the mean. A two-way Scheirer–Ray–Hare test indicated a significant effect in the FR requirement (H_1_ = 14.05, p = 0.0002) and the PR reward volume (H_1_ = 15.72, p = 7 × 10^–5^) with significant pairs notated (post hoc Rank-sum test with Bonferroni correction, **p < 0.01, ***p < 0.001).

We estimated the PR requirement at indifference points by fitting the experimental data to a function that represented the choices. Because our data was binary (i.e. two choices), session data were fit with a sigmoid function (Boltzmann function). Sigmoid fitting curves captured the profiles of mouse decisions, showing the transition from PR to FR (Fig. 2A,B). The indifference trial number was estimated where the sigmoid curve crossed the midline. Then the number of lever presses required for the PR at that trial was extracted from the data, which provided the PR requirement at the indifference point of the session data. Figure 2A also shows where the indifference point lies compared to the increasing PR requirement over time. The results showed that both at a single mouse level and at the animal average, the estimated PR requirement at indifference points was lowest at 2xFR6 and highest at 5xFR12, while 2xFR12 and 5xFR6 had intermediate values (Fig. 2C). We also estimated indifference points using a median of trials that captured the wide distribution of later PR choices, with results presented in Supplementary Fig. S4. The two approaches showed consistent shifting patterns of the PR requirement at the indifference point proportional to the relative values of the PR. This indicates that mice were able to adjust their switching decisions according to the given value.

### The cost parameter contributes more strongly to suboptimality

When is the right moment for mice to switch their preference from the PR to the FR? What is an optimal strategy to maximize gains? How close is mouse behavior to optimal? To answer these questions, we took advantage of optimality models in foraging theory. The long-term rate of gain intake (also referred to as the ratio of expectations or RoE) is often assumed to be the optimal ‘currency’ to maximize because it minimizes the loss of alternative opportunities^27^. An alternative to RoE, which is not theoretically optimal but may better represent the currency that animals actually maximize, is the expectation of ratios (EoR, also called the short-term rate) which measures the average per-trial ratio of gains to costs^27^. The comparison between RoE and EoR analysis in our task is described in Supplementary Appendix.

Because of the trial-based structure of the task, we studied mouse behavior in reference to the EoR model where a rational agent is assumed to maximize the average per-trial rate of benefit over cost. Note that our EoR is a modified version of the original EoR in order to account for the discrete nature of lever presses. EoR is given by the following equation:

1 $$ Expectation \,of \,Ratios \left( {or \,EoR} \right) = \frac{1}{N}\mathop \sum \limits_{k = 1}^{N} \left( { \frac{{r_{k} }}{{p_{k} }} } \right), $$

where *N, r*_*k*_*,* and *p*_*k*_ denote the total number of trials, reward on trial *k*, and cost of lever press on trial *k*, respectively.

**Figure 3:**
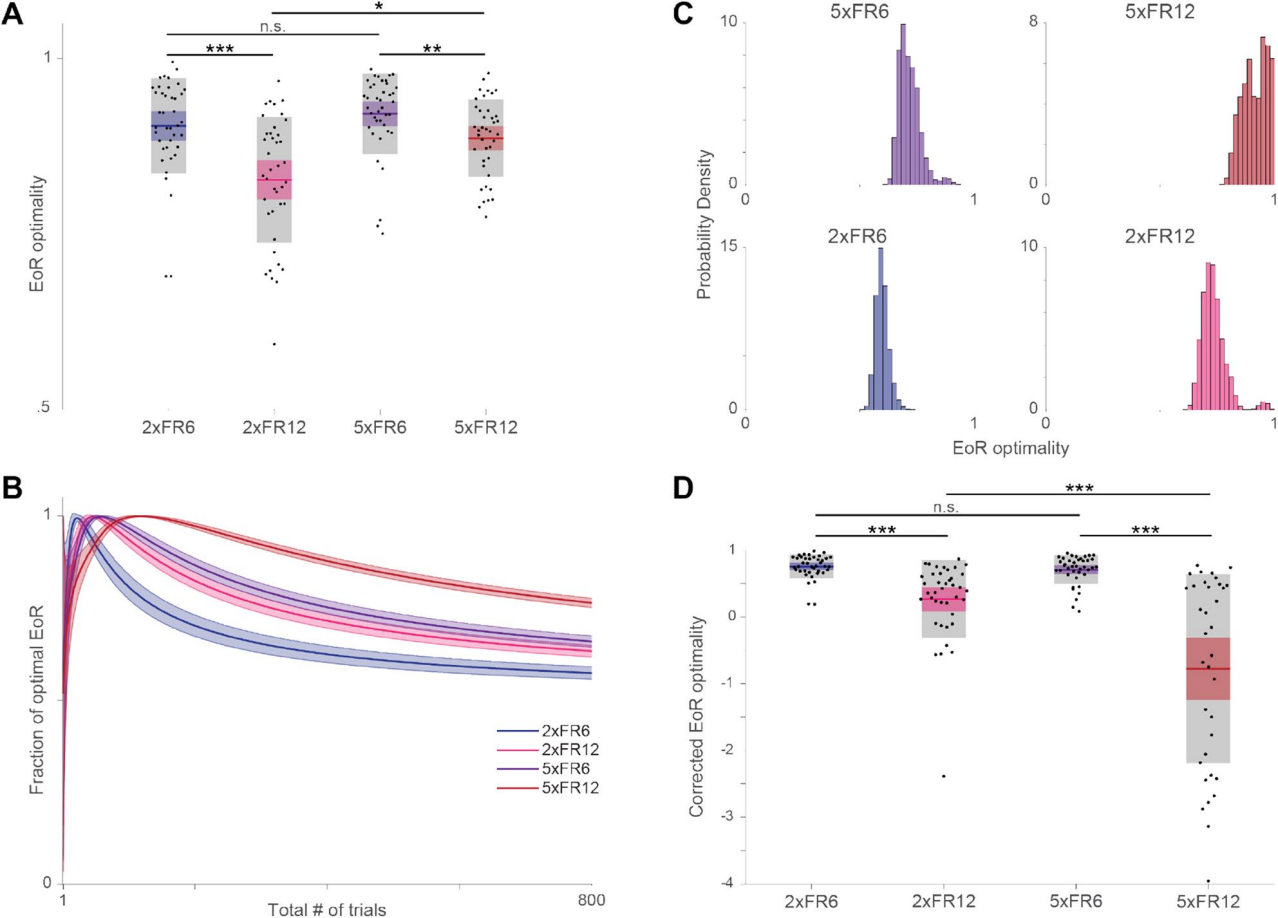
Rate-maximizing (EoR) and random choice models. (**A**) EoR optimality distributions by session type. Each point represents the EoR optimality from one session. Colored line, colored shaded box, and grey box show the mean, 95% confidence interval for the mean, and the standard deviation, respectively. Scheirer-Ray-Hare test indicates a significant effect of both PR reward size and FR lever press requirement on EoR optimality. *p < .05, **p < .01, ***p < .001 indicates significance for post-hoc pairwise two-tailed rank sum tests. (**B**) EoR optimality of a randomly choosing agent for a given number of trials. For a given number of trials (1–800) 10,000 agents that chose randomly between the FR and PR sides were simulated and their fraction of the optimal EoR for that number of trials was recorded. Dark lines indicate the mean and shaded regions show the standard deviation. (**C**) Distribution of EoR optimality for randomly choosing agents given mouse trial statistics. For each session a mouse performed, 10,000 randomly choosing agents were simulated for the number of trials the mouse performed that session. Therefore, each histogram shows the results from 400,000 random agents. Because the domain of each histogram is smaller than 1, probability density of each bin can be greater than 1. (**D**) Corrected EoR optimalities. For each session a mouse performed, it’s observed EoR optimality [shown in (**A**)] was corrected (see Eq. 3) by using the mean EoR optimality of the 10,000 random agents simulated for that number of trials [mean value for appropriate session type and number of trials shown in (**B**)]. Negative values mean the mouse did worse than a random agent and positive means the mouse did better than a random agent. Colored line, colored shaded box, and grey box are as in (**A**). Scheirer–Ray–Hare test again showed significant effect of PR reward size and FR lever press requirement on these corrected values. Significance bars for post-hoc tests are as in (**A**).

Using this equation, we calculated the optimal number of PR trials that the mouse should complete for a given session type, where optimality means maximizing the EoR. The estimated optimal numbers of trials at the PR side ($N_{PR}^{*}$) are 10, 22, 28, and 58 trials at 2xFR6, 2xFR12, 5xFR6, and 5xFR12 respectively (Fig. 3A). Using these numbers, the optimal EoR (EoR^opt^) was calculated and compared with EoR^mice^. The EoR optimality was defined as the ratio of EoR^mice^ over EoR^opt^.

2 $$ EoR \,optimality = \frac{{EoR^{mice} }}{{EoR^{opt} }}. $$

This value lies between 0 and 1, where 1 means mice perform the task as an optimal agent.

The mean EoR optimality was 0.90 ± 0.01, 0.83 ± 0.01, 0.92 ± 0.01 and 0.88 ± 0.01 (mean ± SEM) at 2xFR6, 2xFR12, 5xFR6, and 5xFR12 respectively (Fig. 3A). This reflects that mice are fairly optimal in all conditions, although one noticeable trend was that the change in the cost parameter had a more significant effect on the deviation from the optimality. For instance, given a fixed benefit parameter (2× or 5×), the EoR optimality of FR6 was higher than that of FR12 (Fig. 3A). Given a fixed cost parameter, however, the change in the benefit had less influence on the deviation (Fig. 3A). One explanation of this result is that increasing the cost parameter contributed more to the incomplete trials (Supplementary Fig. S3C–F), which contributed negatively to the rate of reward collection. Note that mice revisited the PR side more for FR12 conditions than FR6, which also contributed to suboptimality by indirectly increasing the number of incomplete trials. In summary, the cost parameter, not the benefit, was one main source that led to the suboptimal outcome.

### Comparing behavioral optimality across experimental regimes

A complication of using EoR as a metric to compare the optimality of behavior across the different experimental parameter regimes is that it is ‘easier’ to achieve a higher fraction of the optimal EoR by purely random behavior in the different regimes (Fig. 3B). That is, by acting purely randomly (i.e. choosing either the FR or PR side with 50% probability on each trial), an agent is highly likely to achieve a higher fraction of the optimal EoR in the 5xFR12 condition than the 2xFR6 condition. Additionally, depending on the total number of trials to be completed, choosing between the PR and FR sides randomly can lead to close to optimal behavior (Fig. 3B,C). The EoR optimality of random choices is 0.60 ± 0.027, 0.73 ± 0.055, 0.72 ± 0.046, and 0.92 ± 0.055 (mean ± SD) at 2xFR6, 2xFR12, 5xFR6, and 5xFR12, respectively, when the total numbers of trials were chosen from the mouse data (Fig. 3C).This shows that acting randomly can lead to close to optimal behavior in some contexts, as long as the agent stops at a certain number of trials (this number is equivalent to 2 × $N_{PR}^{*}$, where $N_{PR}^{*}$ is the optimal number of PR trials for a given experimental parameter set).

We therefore desired to compute an adjusted performance metric that corrects for the differences in optimality achieved through random behavior. To do this, we computed the distribution of EoR optimality values achieved by a random agent for each number of total trials completed from 1 to 1000 trials (see “Methods”). Then, for each session a mouse completed, we compute the following quantity:

3 $$ corrected\, EoR \,optimality = \frac{{EoR_{mouse}^{optimality} \left( n \right) - EoR_{random}^{optimality} \left( n \right)}}{{1 - EoR_{random}^{optimality} \left( n \right)}}, $$

where $EoR_{mouse}^{optimality}$ is the EoR optimality from (2), $EoR_{random}^{optimality}$ is the mean of the distribution of equivalent optimality scores obtained from comparing the EoRs of the random agents to the optimal EoR (Fig. 3B), and $n$ is the number of trials the mouse performed during the session. To explain the corrected EoR optimality by way of example, a value of 0 indicates the mouse performed as well as a random agent, a value of 1 indicates the mouse was optimal, a value of 0.5 indicates the mouse had an EoR optimality equal to $EoR_{random}^{optimality}$ + 50% of the difference between optimal and the random agent EoR optimality, and a value of − 1 indicates the mouse had an EoR optimality equal to $EoR_{random}^{optimality}$ − 100% of the difference between optimal and the random agent EoR optimality.

This corrected EoR optimality achieved by the mouse can then be interpreted as the performance of the mouse above and beyond simple random choice while accounting for the differences in optimality fractions achieved by the random agents in the different session types and thereby provides a corrected quantity to compare across the 4 experimental parameter sets (Fig. 3D, Supplementary Fig. S6). Intuitively, negative values mean the mouse did worse than a random agent and positive means the mouse did better than a random agent. Corrected EoR optimalities were 0.76 ± 0.18, 0.27 ± 0.58, 0.71 ± 0.22, and − 3.89 ± 9.72 (mean ± SD) for the 2xFR6, 2xFR12, 5xFR6, and 5xFR12 sessions respectively (Fig. 3D). The mean and standard deviation reported here for the 5xFR12 sessions are skewed by 5 outliers with corrected EoR values less than − 5 (Supplementary Fig. S6). These resulted from sessions where mice performed fewer than 200 trials (and for 4 out of the 5 outliers, less than 150 trials). Removing these 5 datapoints gives mean ± SD values of − 0.77 ± 1.42 in the 5xFR12 session.

It should be noted that by making this comparison to random behavior (and noting that random behavior can be optimal given the correct number of trials) we are not suggesting that mice adopted a random choice strategy in this task. Mouse behavior was definitively non-random in that mice consistently preferred the PR side early on in the session and shifted to preferring the small reward side as the PR press requirement increased. However, it is worth noting that mice continuously re-visit the PR side to collect large reward, even after passing indifference points (Fig. 1C, Supplementary Fig. S5). This can be viewed as an innate explorative behavior that is useful in an uncertain and changing environment or when an agent has yet to find an optimal strategy in a fixed environment.

### Susceptibility to sunk cost fallacy contributes to suboptimality

Once a resource (e.g., time or effort) has been spent it cannot be recovered. These investments are referred to as sunk costs^28^. An optimal subject would consider only expected future rewards when making decisions, but studies on human decision-making have shown that subjects sometimes commit the sunk cost fallacy, where decisions are made based on irrecoverable sunk costs while neglecting more beneficial options^18^. This phenomenon was originally thought to be unique to humans; however, recent studies have suggested that other animals also consider sunk costs during foraging behaviors^9,19^. In our task, after passing the indifference point, mice often revisited the PR side, which required an unreasonably high number of presses, and keep making presses on the said side until they collect the reward. This observation was similar to what Sweis et al. reported^9^ and led us to investigate whether susceptibility to sunk cost fallacy could be identified and quantified in our dataset.

**Figure 4:**
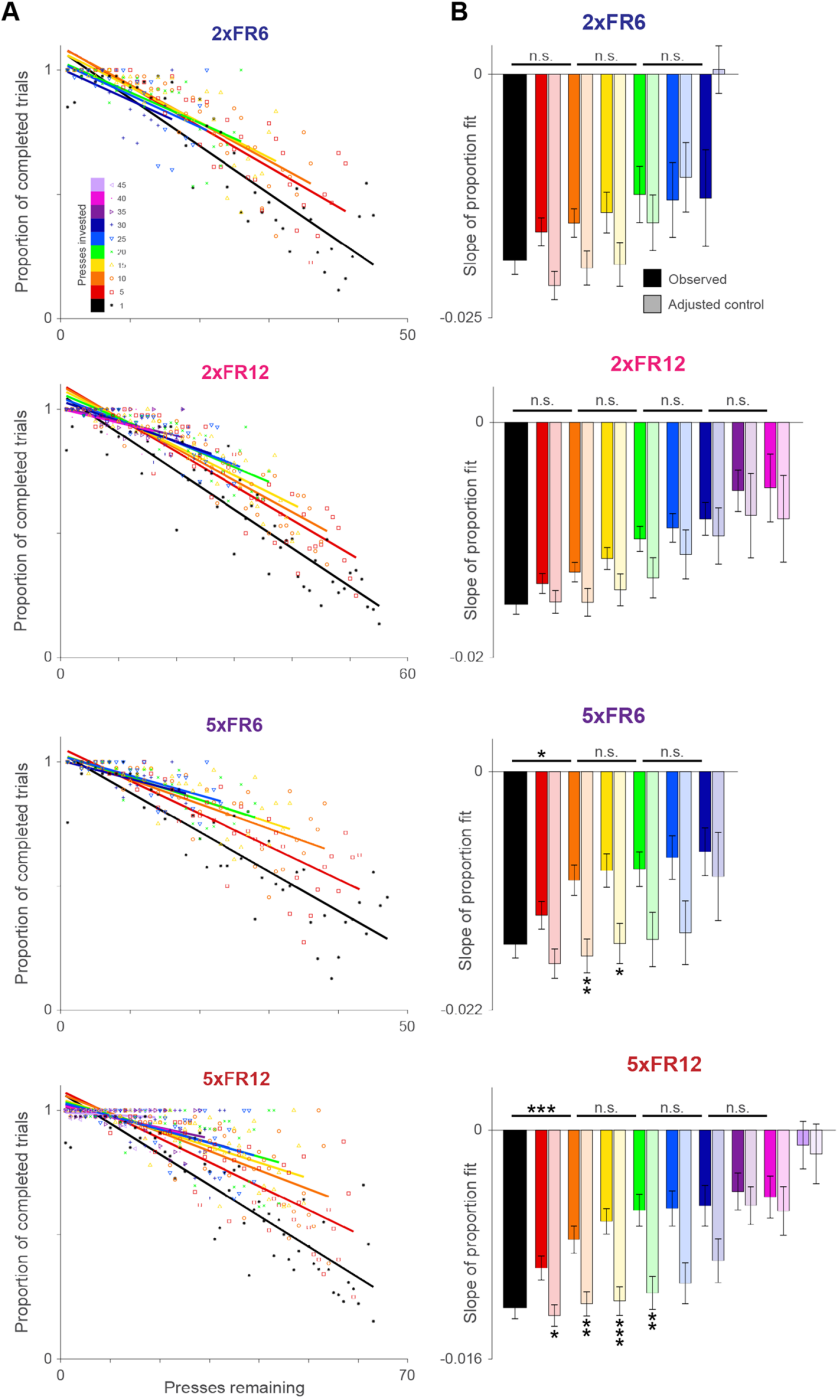
Susceptibility to sunk cost fallacy contributes to the suboptimality. (**A**) Probability of collecting the large reward given how many presses are remaining and how many presses have already been invested. Each color reflects a different number of presses the mouse has already pressed. (**B**) Slopes from each regression in (**A**) (“observed”) and slopes computed from black data points limited to the range of each colored group (“adjusted control”; Supplementary Fig. S7). Error bars reflect standard error of the mean. An analysis of covariance (ANOCOVA) was used to compare slopes of the linear regression models, correcting for multiple comparisons. Not significant (n.s.), p > 0.05; *p < 0.05; **p < 0.01; ***p < 0.001; ****p < 0.0001.

To quantify sensitivity to sunk costs, we examined rewarded and incomplete PR trials. We parameterized the proportion of successful PR trials as a function of the number of presses remaining and the number of presses already invested. Our data allowed us to analyze multiple conditions of presses invested in order to determine the impact sunk costs have on decision-making. For each condition, the proportions of completed trials were fit to a linear function (Fig. 4A). If mice exhibit a susceptibility to sunk cost fallacy in our behavioral task, we would expect higher proportions of completed trials when more presses are invested. This would be reflected by the regression slope approaching zero (Fig. 4B).

The application of this analysis to our dataset revealed that indeed an effect of sensitivity to sunk cost existed. Surprisingly, it is not present in all four conditions of the task. A two-way ANOVA collapsing across sunk cost conditions revealed that the effect was only significant in contexts with a higher cost or benefit parameter (2xFR12: F = 31.8, p < 0.0001; 5xFR6: F = 8.4, p < 0.01; 5xFR12: F = 6.76, p < 0.01) and not in the lowest PR value context (2xFR6: F = 3.1, p = 0.08). The slopes of sunk cost conditions were also compared to neighbors and adjusted controls (Supplementary Fig. S7) to illustrate that sensitivity to sunk costs increased with an increase in investment. Taken together, the results suggest that mice show a susceptibility to sunk cost fallacy in our task, which also contributes to suboptimal performance.

### Optimal and suboptimal regimes in the parameter space

The results thus far indicated that the 2xFR6 condition is different from the rest. Sensitivity to sunk cost did not have much impact on 2xFR6 where the large reward option was particularly unattractive and the small reward cost was low. Our results from Df1/ + mice (a mouse model of 22q11.2 deletion syndrome, the largest known genetic risk factor for schizophrenia) still showed a susceptibility to sunk cost fallacy in the 2xFR6 condition (data not shown), serving as a positive control condition. Both the reduced explorative behavior and susceptibility to sunk cost fallacy in the 2xFR6 are probable cause of high EoR optimality in 2xFR6 condition. In other words, the mice perform almost rationally in this regime.

## Discussion

Applying quantitative behavior and a theoretical framework to a mouse model system, we investigated the factors that contribute to suboptimal outcomes in an economic decision-making behavior. We found that both the cost and benefit differentially contributed to the suboptimality. An increased cost reduced the success rate of reward collection and was the main factor contributing to suboptimality, but the benefit parameter also contributed indirectly via sensitivity to sunk cost. In addition, delineating rational and irrational regimes in the same behavioral task is a unique contribution to the field.

Our task design was inspired by several previous studies spanning the areas of foraging theory, economic decision-making, motivation, and irrationality^17,20–26^. Whether animals are rational or irrational is a controversial and active research question^29^. Several previous studies with laboratory animals reported that foraging decisions often approximate the optimal solution, while others reported to the contrary^3,9,12,16,30^. The various meanings of rationality used in different fields add additional complexity^12,29^. Because the behavioral readout of laboratory animals is contingent on several factors such as internal state, training history, and contexts that are sometimes difficult to control, the results from one study tend to only support one side of the argument. In this regard, a novel contribution of the current study is to provide a behavioral paradigm where both (close to) rational and irrational regimes exist in the same task.

A few things need to be discussed about the potential pitfalls in our task and interpretation of the results. First, although water and food rewards are widely used in animal studies, they are different from non-sating rewards^31,32^. Changes in the motivational level of animals due to satiation are unavoidable. Since motivation is a key factor that shapes the animals’ behavior, caution is needed when interpreting results with satiating rewards. In our task, however, the switching decisions at the indifference points occurred at the beginning of the session. Because the total number of trials that mice perform is an order of magnitude higher, we argue that the sating effect of water reward in our task is negligible. Second, the estimated indifference points in our study only approximate the true equilibrium because we did not test the hysteresis effect on the indifference point. In order to account for the hysteresis effect, the indifference points estimated from the PR to FR switching should be compared to switching from FR to PR. While it is very difficult to test for hysteresis in our task design, according to one study with probabilistic discounting as a cost parameter, hysteresis indeed exists in rat behavior^33^.

Although our choice of the EoR (Expectation of Ratios) as an optimal model led to several important conclusions in the current study^27^, the limitations of this model also need to be discussed. First, according to the EoR, the total number of PR trials completed is an important factor that determines the optimal rate, but the order of choices has no effect. In our switching task, mice almost always stayed on the PR side at the beginning and switched to the FR side as the cost of the PR side increased. The EoR model alone could not account for this behavioral pattern. According to our simulation results (data not shown), however, a simple reinforcement learning model where the reward is replaced with the per-trial ratio of reward to lever presses explains this behavior well. Second, it is easier to achieve a higher fraction of the optimal EoR through random choice for some cost/benefit parameters than others. Therefore, we provide a corrected measure of EoR optimality for comparison across experimental parameters. Third, effort and time cost are different physical entities and thought to be processed in different computational modules in the brain^24,34–37^. The current model neither tried to separate them, nor considered the nonlinearity of time delay and effort cost. These issues need to be addressed in future studies. Fourth, the EoR optimality was estimated under an assumption that costs and benefits linearly contribute to the value of the choice, which is not always true. We believe the linear assumption does not hold at 5xFR12; therefore, it generated a prominent discrepancy between experimentally estimated indifference points and the theoretically estimated optimal number of PR trials. Finally, other models and strategies may exist that potentially explain mouse behavior in the switching task better^3,4,38^. Taken together, although the EoR serves as a useful reference to understanding the mouse behavior in our task, these limitations should be in mind when interpreting the results.

One may argue that the signature of sunk cost fallacy that we observed might simply be an effect of temporal discounting, but we argue against it. According to temporal discounting, the subjective value of the reward increases as mice get closer to receiving a reward by pressing a lever; therefore, the probability of receiving a reward increases accordingly. If it is temporal discounting, this pattern should exist in all conditions that we tested. However, the fact that 2xFR6 condition stands out differently excludes the possibility of temporal discounting.

Valuation is a fundamental cognitive process in decision-making and its dysfunction is expressed in diverse psychopathology including addiction, schizophrenia, depression, anxiety disorders, and severe impulsivity^39–43^. Although it is preliminary, we applied this platform to Df1/ + mice and found behavioral differences compared to wild type. By applying advanced tools in neuroscience, it may be possible to advance the understanding of biological substrates underlying rational and irrational decision factors as well as the function and dysfunction of the neural circuits involved in this process. It will also be interesting to see how mouse models of other diseases behave in our task.

## Materials and methods

### Animals

All animal procedures were performed in accordance with National Institutes of Health standards and were approved by the Brandeis University Institutional Animal Care and Use Committee. Five female and five male mice of the strain C57BL/6 were used in this study. These animals were bred on site from mice purchased from Charles River Laboratories (Wilmington, MA). The mice tested were between the ages of 8 and 13 months. All mice were kept on a 12 h/12 h light–dark cycle.

### Behavioral setup

Experiments were conducted in a rectangular acrylic testing chamber (length, 19.3 cm; width, 14.0 cm; height, 13.9 cm, SanWorks, Stony Brook, NY) with grated flooring. A metal tray was placed underneath the chamber to collect waste. The chamber contained three nose-pokes with infrared LED/infrared phototransistor pairs (Digikey, Thief River Falls, MN) to detect responses. A white LED (Digikey) inside the nose-pokes was used to cue trial availability, lever pressing progress, and reward availability. Only the center port was used for reward delivery. The two end ports were used only as lights and were covered with a snuggly-fit clear plastic cap. Plastic levers were custom designed for us by SanWorks for either side of the nose-pokes. The levers were also equipped with an infrared sensor to capture lever presses. A food pellet was placed inside the testing chamber at the start of each session to allow mice to eat in between trials. The testing chamber was situated inside of a custom-built noise-reducing box (length, 42 cm; width, 39 cm; height, 39 cm). Water reward was delivered through a solenoid valve inside the nose-pokes (Lee Valve Co, Westbrook, CT). Water was supplied by a 60 mL syringe barrel mounted to the inside wall of the box and connected to the valve with silicone tubing (1/16″ × 3/16″, Saint-Gobain Tygon, Malvern, PA). The syringe was refilled after every session to maintain water pressure. Two computer speakers (AmazonBasics, Seattle, WA) were placed inside the box on either side of the testing chamber to deliver punishment sounds. An infrared camera (Logitech, Binghamton, NY) was attached to the top of the inside of the box to allow observation during sessions. The testing chamber was connected to a Bpod state machine (SanWorks). Trial events were triggered through Matlab (MathWorks, Natick, MA).

### Training

Prior to training, animals were water-restricted for 24 h. Water was given daily to maintain 85–90% of their free-drinking body weight. Training occurred in 5 phases and took about 2–3 weeks. For all stages, mice were able to move to the next phase of training on the following day if they performed ~ 80 or more successful trials within an hour. First, mice were placed into the testing chamber to acclimate and could enter the center nose-poke (indicated by the center nose-poke being lit) for a small water reward (4 µL). The light turned off when the mouse entered the port and collected the reward. After the mouse exited the port, there was a one second delay before the next trial began. Second, mice had to press the right lever once (indicated by the right nose-poke being lit) for the center nose-poke to light up and provide reward. The lever had to be depressed for at least 100 ms to register as a press. Third, mice repeated the second phase but on the left side. Fourth, the animal had to press either the left or right lever once to obtain reward. The trials were pseudo-randomized and the correct side to obtain reward was indicated by the corresponding nose-poke being lit. Finally, mice repeated the fourth phase, but with an increasing number of presses required each day, from 2 presses all the way up to 10 presses. Once mice completed these stages of training, they were subjected to the optimal switching task and could familiarize themselves with the task for 3–5 sessions before data collection began.

### Switching task

Our behavioral task involved combining a progressive ratio (PR) and fixed ratio (FR) schedule of lever pressing. The PR was associated with a large volume of water (either 6 µL or 15 µL) and the FR was associated with a small volume of water (3 µL). In addition, the FR could either be 6 presses or 12 presses. Similar to the training phases, levers had to be depressed for 100 ms to count as a press and there was a one second delay between trials. The PR and FR sides as well as the parameter pairs were pseudo-randomly chosen at the start of each session. At the start of a trial, both the left and right nose-pokes were dimly lit, indicating that the mouse could choose either the left or right side. Once a mouse chose a side, the corresponding nose-poke would get increasingly brighter with each press until the required number of presses was met and the center port lit up to indicate reward availability. Mice were able to freely choose either side; however, if they decided to switch sides in the middle of a trial before completing the number of presses on the initially chosen side, a punishment sound of white noise would play and the trial would end. That trial was then classified as an incomplete trial. Furthermore, if a mouse began pressing a lever but then stopped for more than 10 s to groom itself, eat, etc., the trial would end and that trial would be considered incomplete. A session could be anywhere from 1 to 3 h long. A session ended if a mouse did not press either lever for a period longer than 5 min. If this did not occur within 3 h, the session was ended by the operator. In this way, we can ensure that the mouse is well sated by the end of the session and that we capture the highest number of trials the mouse is willing to perform without keeping the animal in the chamber too long. At the end of each session, the mouse was weighed, and additional water was given at the end of each session if necessary to maintain the animal’s weight at 85–90% of their free-drinking body weight.

### Biological vs. technical replication

We evaluated an equal number of five male and five female mice and did not see significant differences (Supplementary Fig. S1); therefore, we combined the data for further analysis. Four sessions with each parameter setting were collected (160 sessions total: 4 sessions × 4 parameter pairs × 10 mice). Therefore, our biological replicates are 10 mice and technical replicates are 4 sessions for each parameter settings.

### Data analysis

All data analysis was carried out using built-in and custom-built software in Matlab (Mathworks). The identification of indifference points was done by fitting our binary data to a sigmoid function, the Boltzmann function.

$$ f\left( x \right) = \frac{1}{{1 + e^{{\frac{{\left( {x - x_{0} } \right)}}{\tau }}} }}, $$

where $x_{0}$ and $\tau$ are a 50% threshold and a slope, respectively. By assigning the value of the PR choice = 1 and that of the FR = 0, and assuming that the curve started from the PR and ended to the FR, the fitting curve was generated. An indifference trial number was estimated where the sigmoid curve crossed the midline and the number of lever presses required at the trial on the PR side was extracted from the data. This required number of lever presses provided the PR requirement at the indifference point of the session data. Statistical significance among four parameter pairs was first tested with the two-factor nonparametric Scheirer-Ray-Hare test and the post-hoc pairwise comparison was done with Rank-sum test with Bonferroni correction for 4 comparison pairs. For single pair comparisons, a nonparametric Rank-sum test was used. A *P-value* cutoff of 0.05 was used for significance testing.

### Analysis of sensitivity to sunk cost

Analysis of sensitivity to sunk cost started by taking all attempted PR trials in a session and separating by the number of presses remaining after the mouse made one press. We then determined how many trials in each group were completed. All trials from sessions of the same parameter setting were totaled in each mouse, then averaged across mice. To prevent data from being skewed by a single trial, data points were only included if there were more than five completed trials in that condition. The proportion of completed trials for each value of remaining presses was calculated by dividing the average number of completed trials by the average total number of trials. These proportion values were then fitted linearly. All PR trials were sampled again after 5, 10, 15, 20, 25, 30, 35, 40, and 45 presses had been invested and the respective proportions of rewarded trials were calculated and fitted to new regression lines as described above. Regressions were only included in analysis if there were at least 15 data points being fitted. The overall effect of sensitivity to sunk cost in each context was quantified using a two-way ANOVA with the probability as the dependent variable and number of presses remaining x sunk cost groups as factors. Post-hoc comparisons were done between the regression slopes of investment groups as well as each slope to an adjusted control slope (Supplementary Fig. S7) using an analysis of covariance (ANCOVA) with p-values adjusted for multiple comparisons.

### Calculation of EoR optimality

In past foraging literature, two “currencies” have been proposed as quantities that animals try to maximize as a proxy for their evolutionary fitness^27^. These are the long-term rate of energy intake (also called the ratio of expectations, RoE) and the short-term rate (also called the expectation of ratios, EoR). In terms of total energy intake over time, RoE is the theoretically best quantity to maximize; however, past experiments with foraging starlings have highlighted EoR as the currency that may actually be used^27^. We therefore computed the optimal strategy for this task in terms of EoR and compared it to the mice’s behavior. For our task, the EoR for a given number of trials $N$ is:

$$ EoR\left( N \right) = \frac{1}{N}\mathop \sum \limits_{k = 1}^{N} \frac{{r_{k} }}{{p_{k} }}, $$

where $N$ is the total number of trials, $r_{k}$ is the reward (in µl) received on trial $k$, and $p_{k}$ is the number of lever presses done on trial $k$. Note that this is different than the typical EoR discussed in past studies since the denominator of each fraction is in terms of an energetic cost instead of a time cost. For each session type (2xFR6, 2xFR12, 5xFR6, 5xFR12), we are interested in calculating the optimal number of trials at the progressive ratio ($N_{PR}^{*}$). To do this we want to find which $N_{PR}$ gives us the highest $EoR$ for a given $N$. We can find this by splitting the above equation for $EoR$ up into terms for trials at the PR and trials at the FR.

$$ EoR\left( N \right) = \frac{1}{N}\left( {r_{PR} \times \mathop \sum \limits_{k = 1}^{{N_{PR} }} \frac{1}{k + 1} + N_{FR} \times \frac{{r_{FR} }}{{p_{FR} }}} \right), $$

where $r_{PR}$ is the reward from the PR side, $r_{FR}$ is the reward from the FR side, $p_{FR}$ is the number of lever presses required for reward at the FR, and $N_{FR}$ is the number of completed trials at the FR side. Note that we assume that an optimal agent does not abort any trials since this would strictly decrease the $EoR$ towards 0. Substituting $N_{FR} = N - N_{PR}$ and evaluating the sum we get

$$ EoR\left( N \right) = \frac{1}{N}\left( {r_{PR} \times \left( {\psi^{\left( 0 \right)} \left( {N_{PR} + 2} \right) + \gamma - 1} \right) + \left( {N - N_{PR} } \right) \times \frac{{r_{FR} }}{{p_{FR} }}} \right), $$

where $\psi^{\left( 0 \right)} \left( x \right)$ is the digamma function and $\gamma$ is the Euler–Mascheroni constant. To find $N_{PR}^{*}$ we take the derivative of this expression with respect to $N_{PR}$

$$ \frac{dEoR\left( N \right)}{{dN_{PR} }} = \frac{1}{N}\left( {r_{PR} \times \psi^{\left( 1 \right)} \left( {N_{PR} + 2} \right) - \frac{{r_{FR} }}{{p_{FR} }}} \right), $$

where $\psi^{\left( 1 \right)} \left( x \right)$ is now the trigamma function (derivative of digamma). Setting the derivative to 0 and solving for $\psi^{\left( 1 \right)} \left( {N_{PR} + 2} \right)$ we get

$$ \psi^{\left( 1 \right)} \left( {N_{PR}^{*} + 2} \right) = \frac{{r_{FR} }}{{r_{PR} \times p_{FR} }} $$

from which we can get numerical approximations for $N_{PR}^{*}$ which are 10.5, 22.5, 28.5, and 58.5 at 2xFR6, 2xFR12, 5xFR6, and 5xFR12, respectively. These numbers have a straightforward interpretation. For example, on the 10th PR trial in a 2xFR6 session, the mouse will receive 6 µl of water for 11 lever presses which still gives a better ratio than the FR side (3 µl for 6 presses). On the 11th PR trial these ratios will be equal (6/12 and 3/6). This pattern is true for all 4 session types. Therefore the 0.5 on each of the above $N_{PR}^{*}$ reflects that fact that it is equivalent to stop going to the PR side either when the ratios become equal or 1 trial before. For the analysis in this paper, we took the optimal $N_{PR}^{*}$ for each session type respectively to be 10, 22, 28, and 58. To then calculate each mouse’s fraction of EoR optimality for each session, we calculated the mouse’s observed EoR for that session (mean of per-trial reward divided by the number of presses, aborted trials included) and divided that by the optimal EoR given the number of total trials the mouse performed (including aborted trials) and the session type (Fig. 3B).

### Comparison to random behavior

To compute optimality distributions produced by random behavior for each experimental parameter set (Fig. 3A,C), we simulated 10,000 randomly choosing agents for each number of total trials from 1 to 1000. That is, for each number of total trials from 1 to 1000, we simulated 10,000 agents for that number of trials. On each simulated trial, there was a 50/50 chance of choosing the FR or PR side. The observed EoR of random choices for a given number of trials N was then compared to the optimal EoR for that number of trials to get a fraction of EoR optimality of random behavior $\left( {EoR_{random}^{optimality} \left( n \right)} \right)$. This produced, for each number of total trials, a distribution (from 10,000 samples) of EoR optimalities achieved by simple random choice. To then compare the mice’s performance to random behavior, for each session we computed the corrected EoR optimalities described by Eq. (3). In Eq. (3), $EoR_{random}^{optimality} \left( n \right)$ is taken to be the mean of the distribution of random agent optimalities for the number of trials the mouse completed that session $\left( n \right)$. These ratios quantify the enhanced outcome of mouse choices compared to random choices for a particular experimental parameter set and number of trials (Fig. 3D, Supplementary Fig. S6).

## Supplementary Information

Supplementary Video 1.

Supplementary Video 2.

Supplementary Information.
