# Supplementary material for: Parametric shift from rational to irrational decisions in mice
Source: Sci Rep. 2021 Jan 12;11:480. doi: 10.1038/s41598-020-79949-w (PMC7803778; doi:10.1038/s41598-020-79949-w)
Supplement: Supplementary file 3 — Supplementary Information. [file 41598_2020_79949_MOESM3_ESM.docx]

**Supplementary Information for­­**

Parametric shift from rational to irrational decisions in mice

Nathan A. Schneider, Benjamin Ballintyn, Donald Katz, John Lisman, Hyun-Jae Pi ^*^

*Correspondence: Hyun-Jae Pi ([hyunpi@brandeis.edu](mailto:hyunpi@brandeis.edu))

**This PDF file includes:**

Supplementary text

SI References

Figures S1 to S7

Tables S1

Legends for Movies S1 to S2

**Other supplementary materials for this manuscript include the following:**

Movies S1 to S2

Supplementary Information Text

*RoE analysis and comparison to EoR*

The “long-term rate” or the “ratio of expectations” (RoE)^1^ is defined as:

(1)

$$RoE= \frac{\sum_{k=1}^{N} G_{k}}{\sum_{k=1}^{N} T_{k}} ,$$

where $G_{k}$ is the “energy gain” from the $k$th food item and $T_{k}$ is the time spent acquiring the k’th food item. For this task, we replace $G$ with the size of the reward (in microliters, µl) and $T$ with the number of lever presses required. The index $k$ will now index the trial number.

To get the optimal behavior from an RoE perspective, we calculate the optimal distribution of FR and PR lever presses, where optimality means acquiring as much reward as possible given a certain number of presses. We start by writing down an equation for the reward $R(N)$ acquired:

$R\left( N \right)= N_{FR}\left( \frac{r_{FR}}{P_{FR}} \right)+f\left( N_{PR} \right)*r_{PR} ,$

(2)

where $N_{FR}$ and $N_{PR}$ are the number of presses at the fixed and progressive levers in the session, respectively, the total number of presses $N=N_{FR}+N_{PR}$, $r_{FR}$ and $r_{PR}$ are the FR and PR reward sizes, respectively, $P_{FR}$is the required number of presses at the FR lever, and $f(N_{PR})$ is the number of trials at the PR lever corresponding to $N_{PR}$. Note that we assume the optimal strategy does not contain any aborted trials since an aborted trial will always push RoE towards 0. To start, we need to figure out what $f(N_{PR})$ is. To do this, we set up the following equality

$$N_{PR}=\sum_{k=1}^{f(N_{PR})} k+1 ,$$

expressing that the number of PR presses increases by one with each additional successful PR trial. We then evaluate the sum and solve for $f(N_{PR})$.

$$0={f(N_{PR})}^{2}+3f\left( N_{PR} \right)-2N_{PR}$$

$f\left( N_{PR} \right)=\frac{1}{2}\left( \sqrt{8*N_{PR}+9}-3 \right)$

(3)

Here, we ignore the other root since it is strictly negative. We now plug Equation 2 into Equation 3 and replace $N_{FR}$ with $N-N_{PR}$:

$$R\left( N \right)=\left( N-N_{PR} \right)\left( \frac{r_{FR}}{P_{FR}} \right)+\frac{1}{2}(\sqrt{8*N_{PR}+9}-3)*r_{PR}$$

Now we want to take the derivative of $R\left( N \right)$ with respect to $N_{PR}$ and solve for the optimal number of PR presses ($N_{PR}^{*}$) that will give us $R^{*}(N)$ (the maximum reward achievable given N lever presses).

$$\frac{dR(N)}{dN_{PR}}=\frac{2PR}{\sqrt{8*N_{PR}+9}}-\frac{FR}{P_{FR}}$$

Setting the derivative equal to 0 and solving for $N_{PR}$ gives:

$N_{PR}^{*}=\frac{1}{8}\left( \left( \frac{2PR*P_{FR}}{FR} \right)^{2}-9 \right)$

(4)

Note that Equation 4 yields an $N_{PR}^{*}$ that is independent of N.

For the 2xFR6 case (FR = 3, PR = 6, $P_{FR}$ = 6) this yields $N_{PR}^{*}$= 70.875 presses, $f\left( N_{PR}^{*} \right)$ = 10.5 trials. For the 2xFR12 case (FR = 3, PR = 6, $P_{FR}$ = 12) this yields $N_{PR}^{*}$ = 286.875 presses, $f\left( N_{PR}^{*} \right)$ = 22.5 trials. For the 5xFR6 case (FR = 3, PR = 15, $P_{FR}$ = 6) this yields $N_{PR}^{*}$ = 448.875 presses, $f\left( N_{PR}^{*} \right)$ = 28.5 trials. For the 5xFR12 case (FR = 3, PR = 15, $P_{FR}$ = 12) this yields $N_{PR}^{*}$ = 1798.875 presses, $f\left( N_{PR}^{*} \right)$ = 58.5 trials.

While both potential currencies give the same answer (to within numerical error), we chose to use EoR in this study for several reasons. First, past studies have highlighted EoR as the currency that animals may actually use ^1,2^. Second, most of our analyses focus on single trials rather than individual presses. As a result, we felt the trial-based EoR was more complementary to our other analyses than the press-based RoE. Third, many of our computational models could only be carried out using EoR. Taken together, we believe that EoR is the best theoretical currency for this study.

*Estimation of lever press cost*

In the 2xFR6, 2xFR12, and 5xFR6 conditions mice over chose the PR side (i.e. they completed more than $N_{PR}^{*}$PR trials) but under chose in the 5xFR12 condition where $N_{PR}^{*}$ is highest. We speculated this might be due to an increasing subjective cost per lever press as the number of consecutive lever presses increases. We therefore tried to capture this effect by measuring the inter-press-interval (IPI) as a function of the position of a lever press in a sequence (Fig. S2). However, we found that there was no visible effect until after approximately the 60^th^ consecutive lever press, at which point IPIs began to increase rapidly. We therefore conclude that fatigue due to many consecutive lever presses is unlikely to be the factor that drove under-choosing behavior in the 5xFR12 condition.

**SI References**

1 Bateson, M. K., A. Rate currencies and the foraging starling: the fallacy of the averages revisited. *Behavioral Ecology* **7**, 341-352 (1995).

2 Bateson, M. & Kacelnik, A. Preferences for fixed and variable food sources: variability in amount and delay. *J Exp Anal Behav* **63**, 313-329, doi:10.1901/jeab.1995.63-313 (1995).

| 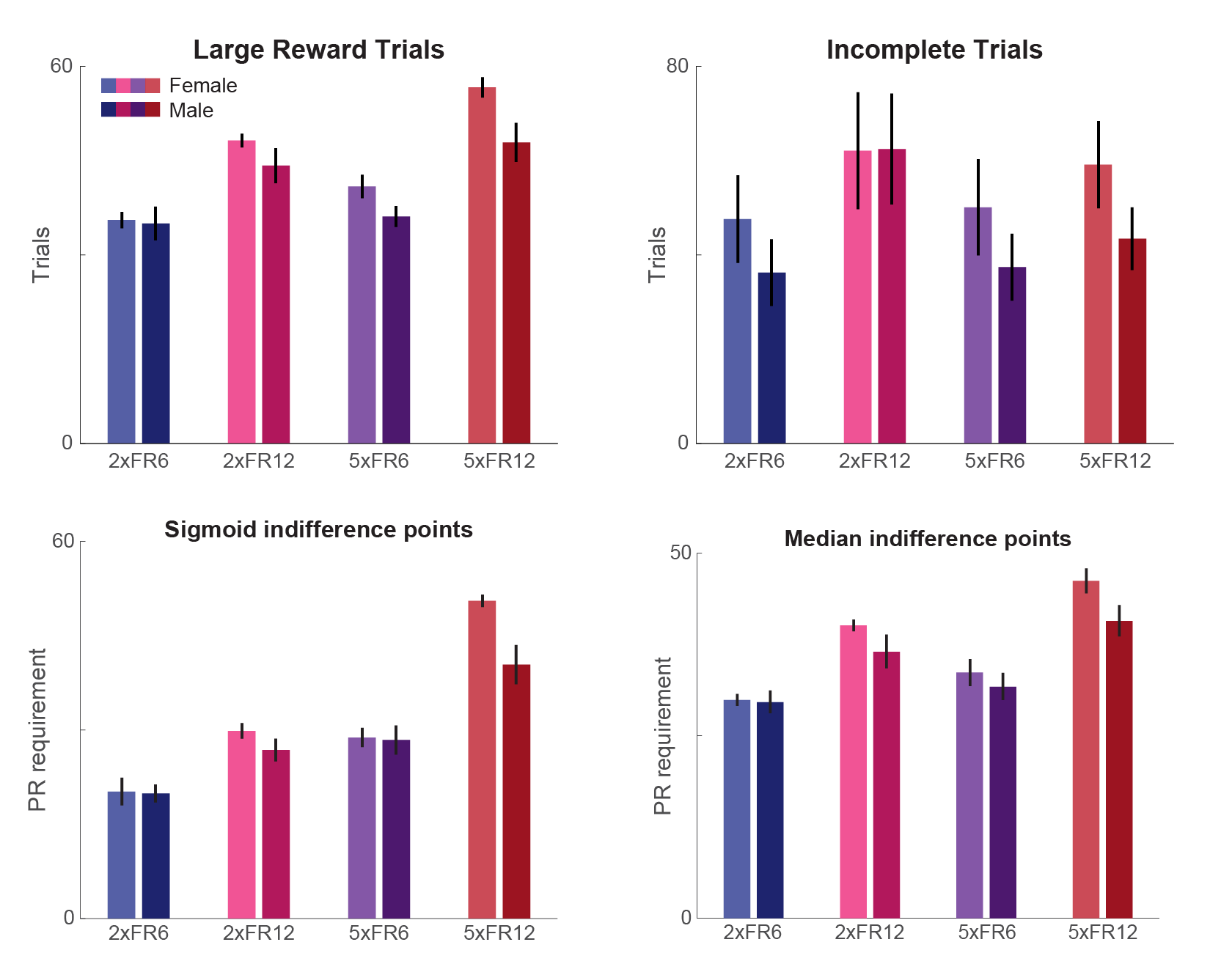 |
| --- |
| **Fig. S1. Comparison of male and female behavior in various contexts.** Five male and five female mice were compared for behavioral differences, and while some measures approached significance in the 5xFR12 condition, no significant differences were identified (Rank-sum test; [Large Reward] 5xFR12, p = 0.06; [Incomplete] 5xFR12, p = 0.21; [Sigmoid] 5xFR12, p = 0.06; [Median] 5xFR12, p = 0.08). As a result, male and female mice were grouped together for all other analyses. Error bars reflect standard error of the mean. |

| 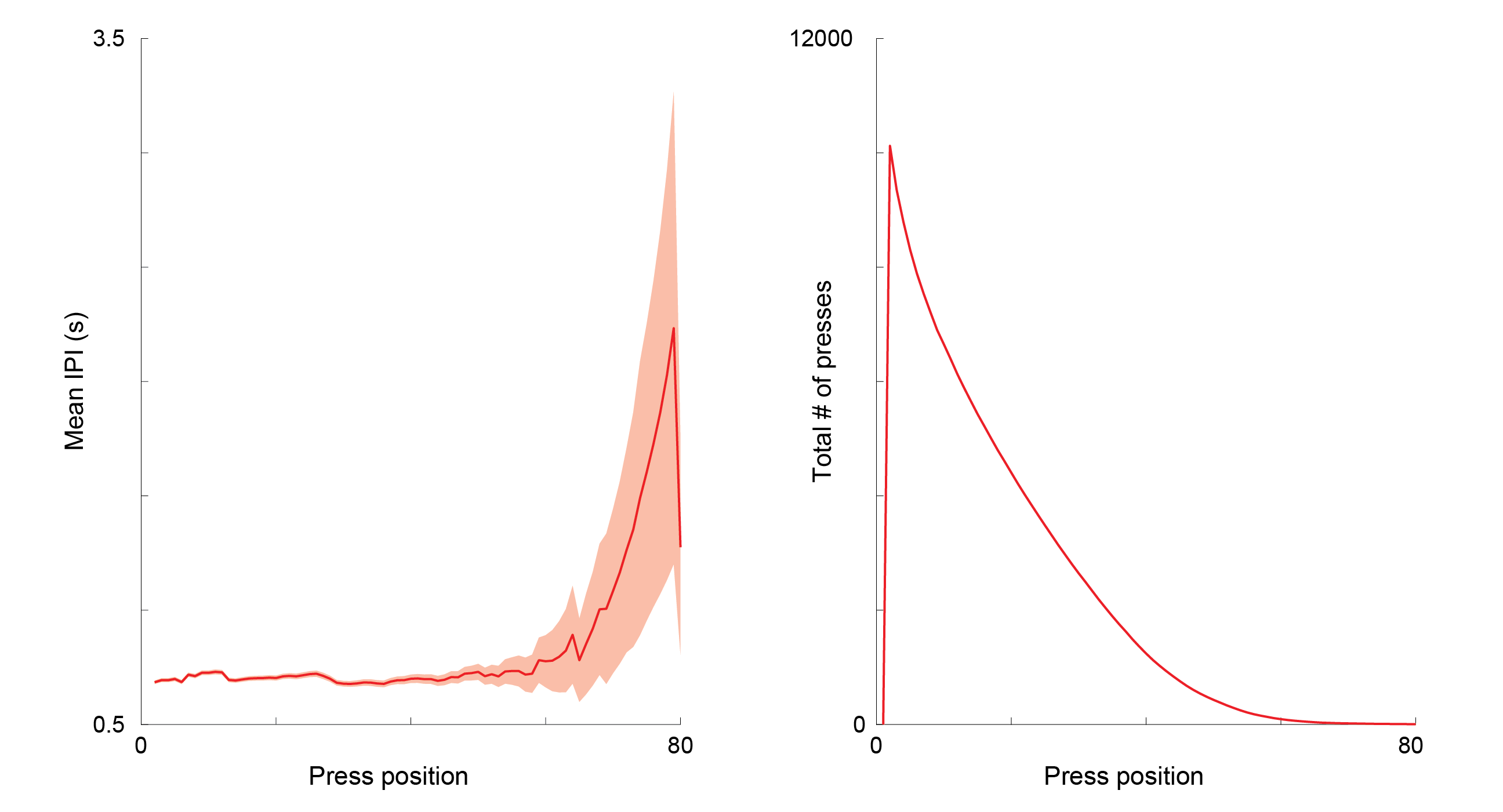 |
| --- |
| **Fig. S2. Inter-press interval (IPI) analysis.** IPI analysis was performed to check whether mice become tired as the number of required presses increases. IPIs (the time between lever presses) were plotted as a function of each lever press’ position in a sequence of lever presses (i.e. do mice take longer on their 50^th^ lever press vs. their 5^th^). We found that while there is some variability in IPIs with increasing lever presses, there is not a significant increase in IPIs until after ~60 lever presses. This suggests that mice are not getting tired and the ‘cost’ of each successive lever press does not increase significantly. Shaded region shows standard error of the mean. Note that press position 80 only contains 5 data points. A sudden drop of mean IPI at position 80 (left panel) was caused by two data points that happened to be much lower (potential outliers). |

**
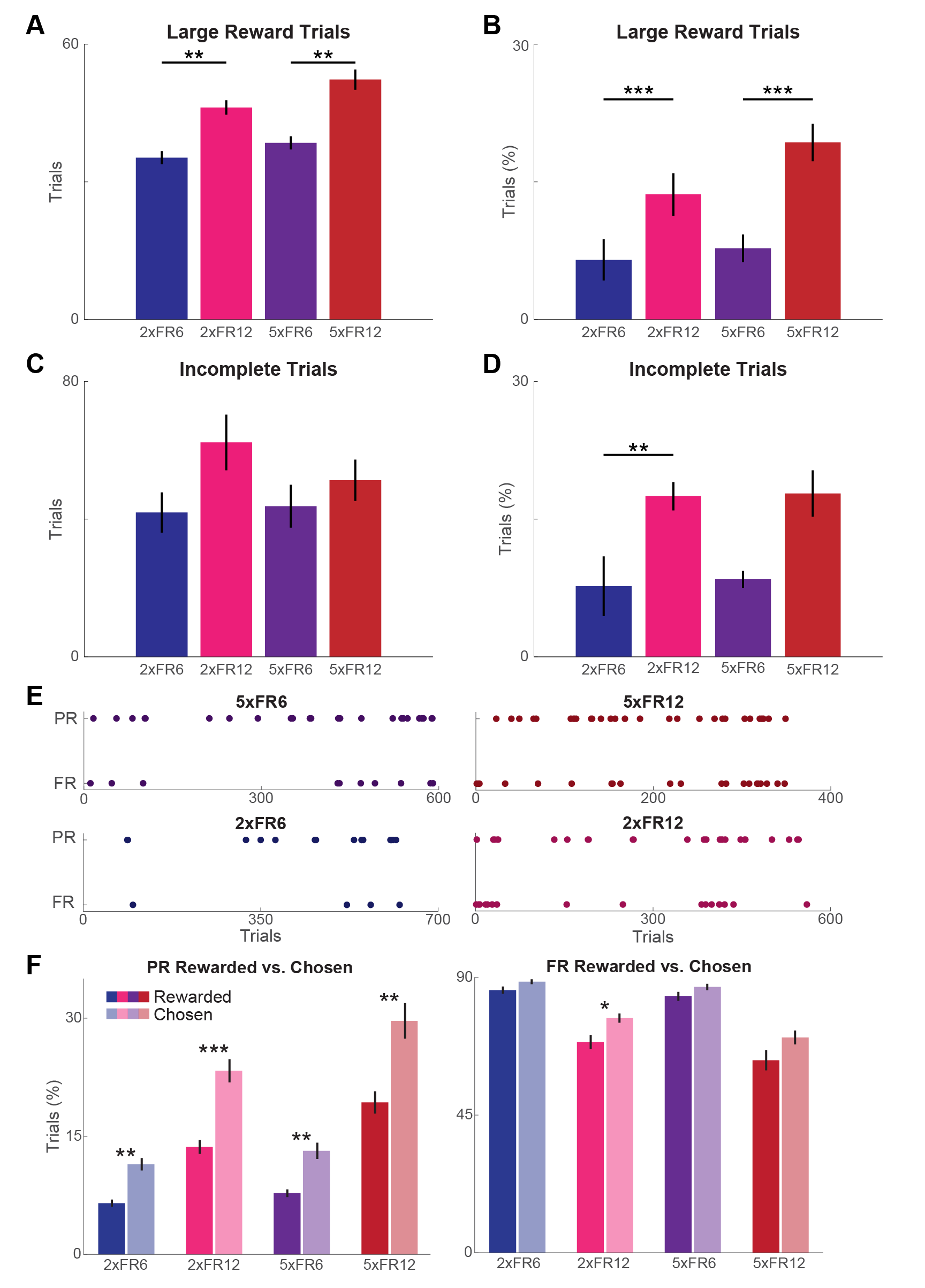
Fig. S3. Contributions of PR and incomplete trials in various session types. A.** Average number of PR trials per session. Error bars reflect standard error of the mean. A two-way Scheirer-Ray-Hare test indicated a significant effect in the FR requirement (H_1_ = 22.56, p = 2 x 10^-6^) with significant pairs notated (post hoc Rank-sum test with Bonferroni correction, **p < 0.01). **B.** Number of PR trials per session normalized to the percent of total trials in the session. Error bars reflect standard error of the mean. A two-way Scheirer-Ray-Hare test indicated a significant effect in the FR requirement (H_1_ = 28.98, p = 7 x 10^-8^) with significant pairs notated (post hoc Rank-sum test with Bonferroni correction, ***p < 0.001). **C.** Average number of incomplete trials per session. Error bars reflect standard error of the mean. No significant differences were detected. **D.** Number of incomplete trials per session normalized to the percent of total trials in the session. Error bars reflect standard error of the mean. A two-way Scheirer-Ray-Hare test indicated a significant effect in the FR requirement (H_1_ = 16.46, p = 5 x 10^-5^) with significant pairs notated (post hoc Rank-sum test with Bonferroni correction, **p < 0.01). **E.** Incomplete trials occur more frequently in the latter half of sessions. Shows example sessions for each parameter pair. Each point is a single incomplete trial within the session. All sessions are from the same animal. **F.** Mice are more likely to attempt and fail when pressing PR. Incomplete trials occur when attempting both the PR and FR. However, there is a significantly larger difference between the percentage of trials attempted on the PR and those actually rewarded (*p < 0.05, **p < 0.01, ***p < 0.001, Rank-sum test, n = 10 mice). Error bars reflect standard error of the mean.

| 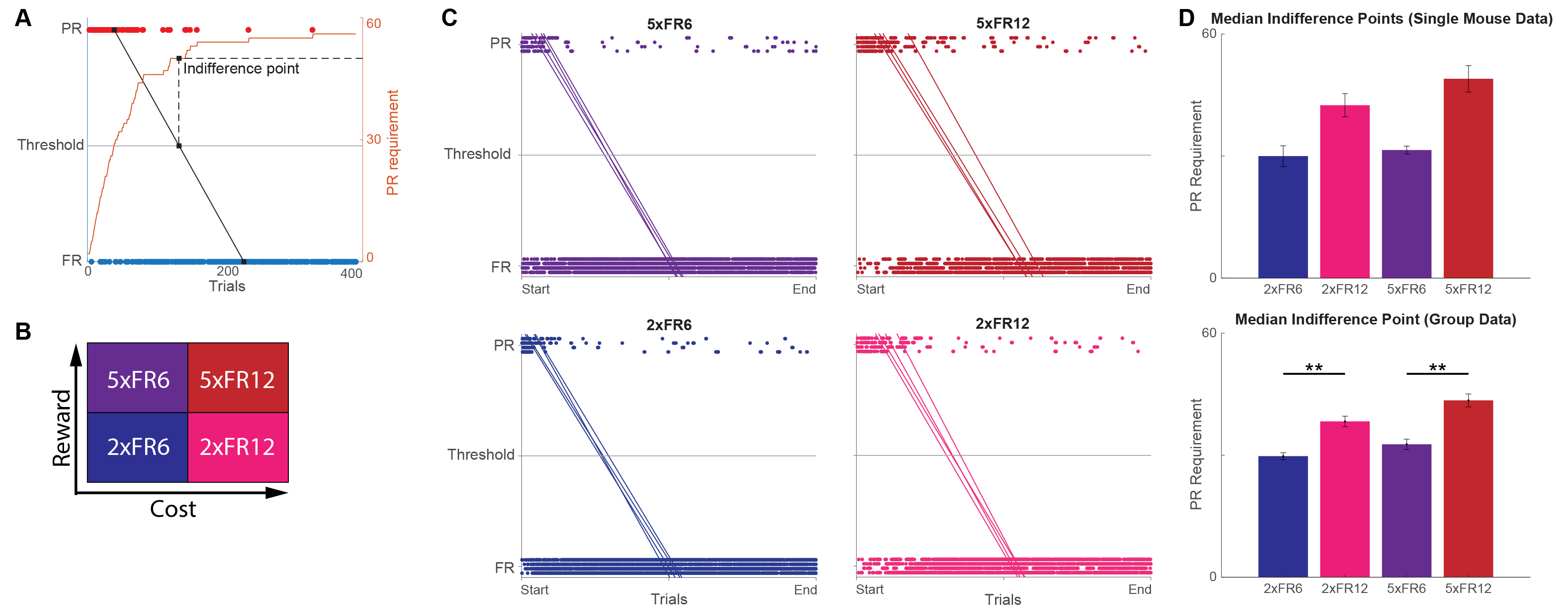 |
| --- |
| **Fig. S4. Calculating indifference points using median trial values displays variation in switching behavior between parameter conditions. A**. Example session showing how the indifference point is estimated. Black line is the linear fit and orange curve is the cumulative PR requirement over time. First, the median trial numbers for PR and FR trials are calculated and connected by a line. Then, where the line crosses the threshold, a trial number is identified (black squares). Finally, the indifference point is determined by finding the number of presses required for the PR at this trial (dashed lines). **B**. Grid showing increasing reward and cost for each type of parameter pair. **C**. Median lines for all 16 sessions of one mouse, sorted by session parameters. Trials are plotted as individual points normalized to the total number of trials in the session and each row of points is a different session. **D**. Indifference points for a single mouse (top, n = 4 sessions) and the entire population (bottom, n = 10 mice). Error bars reflect standard error of the mean. A two-way Scheirer-Ray-Hare test indicated a significant effect in the FR requirement (H_1_ = 22.70, p = 2 x 10^-6^). Insert shows the same data with significant pairs notated (post hoc Rank-sum test with Bonferroni correction, **p < 0.01). |

| 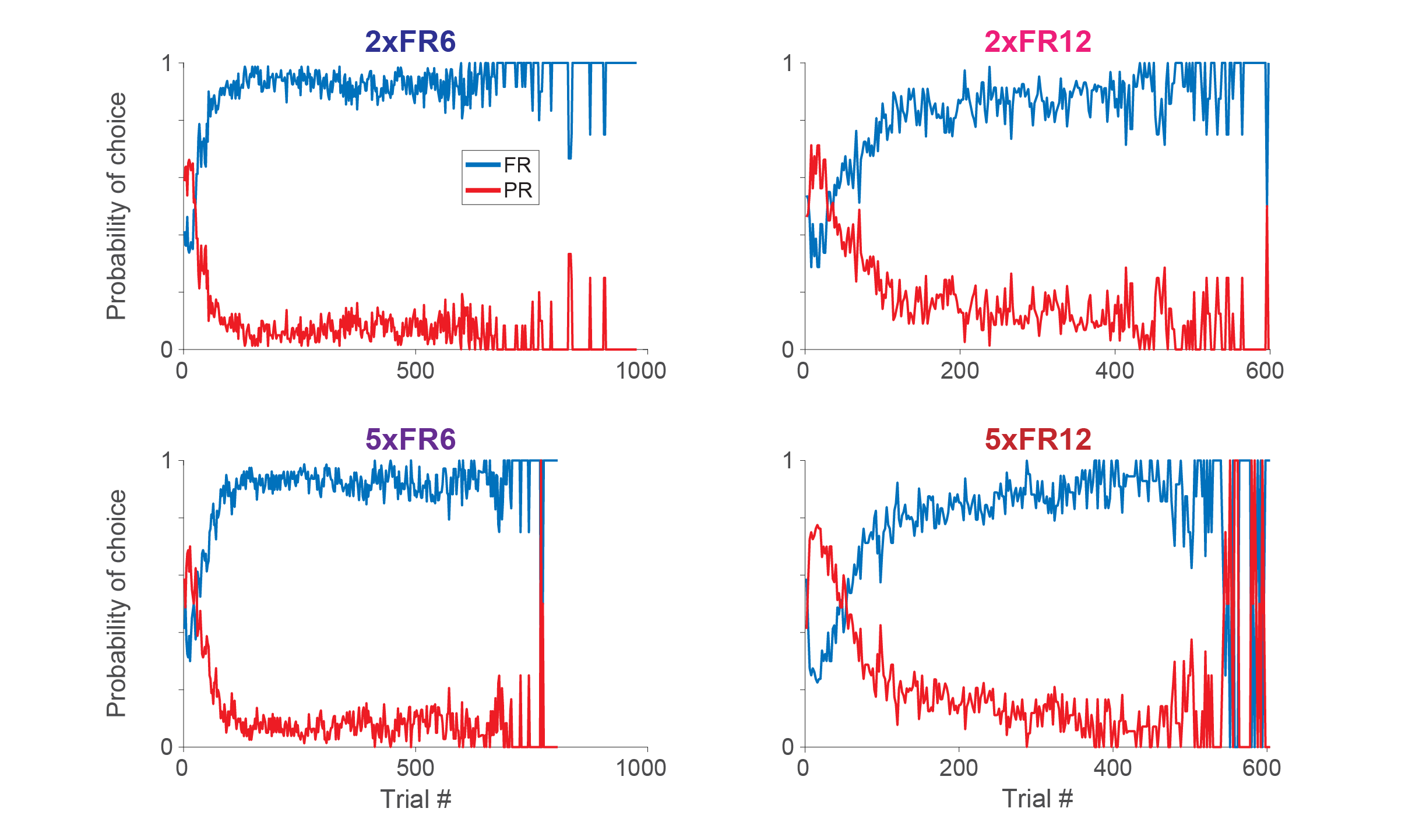 |
| --- |
| **Fig. S5. Probability of choosing the PR or FR side.** Even after passing the indifference points, mice continued to revisit the PR side. These plots visualize the probability of revisiting the PR in different parameter settings. Traces shown here have been smoothed by plotting the mean of each consecutive pair of trials. |


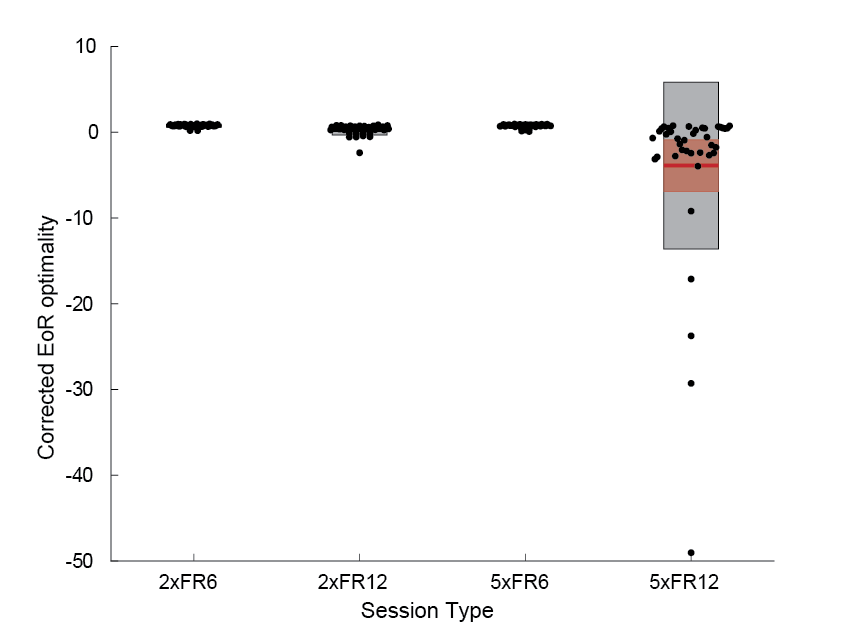


**Fig. S6. Corrected EoR optimalities by session type including outliers.** Layout is identical to Fig. 3D however the full range of values is shown. Outlier points in the 5xFR12 sessions come from sessions where mice completed few trials (<200).

| 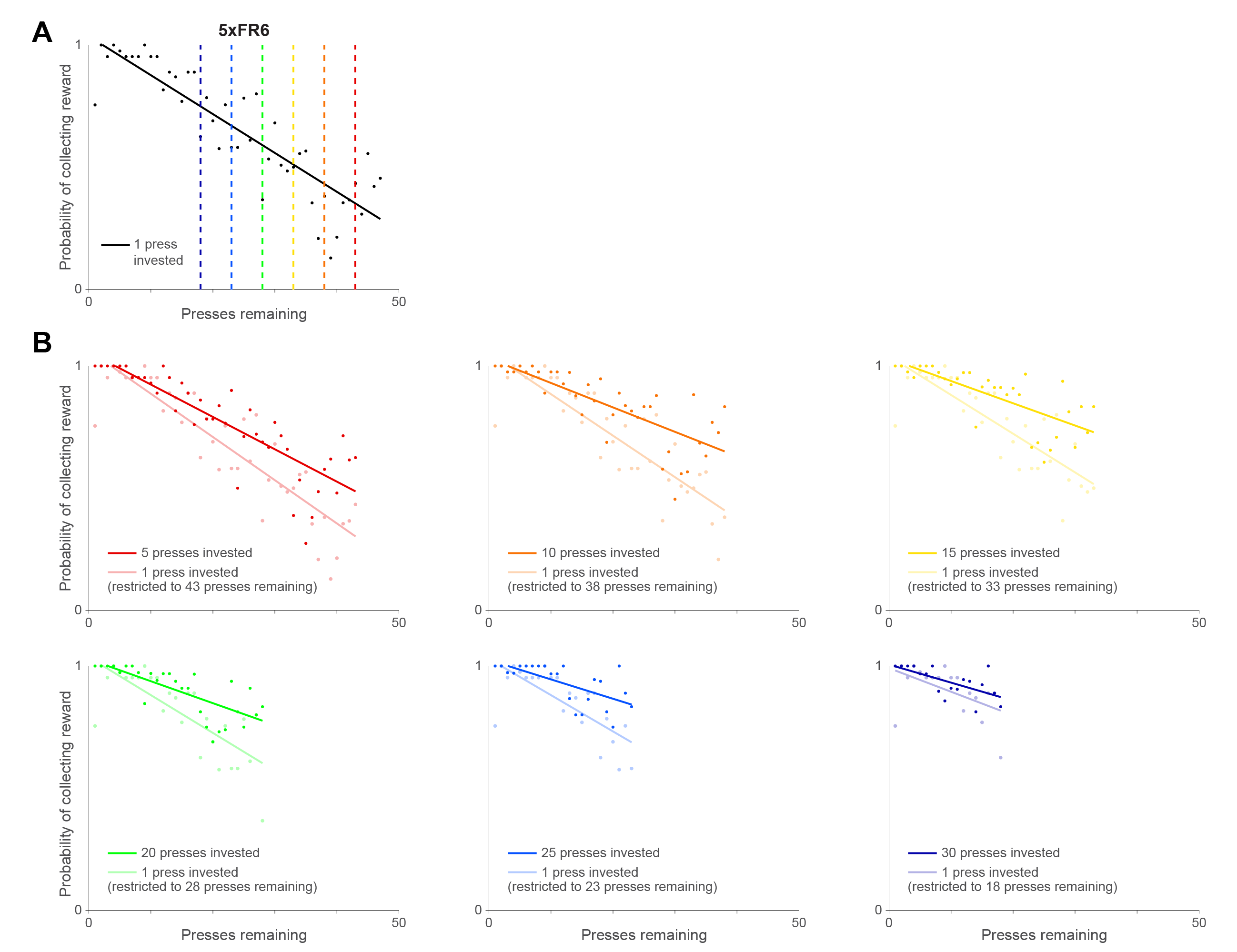 |
| --- |
| **Fig. S7. Visual representation of adjusted control comparisons in sunk cost analysis.** In order to correct for variations in the amount of data available for each sunk cost group, we calculated an “adjusted control” to which we could compare each group. This consisted of taking the data points of the 1 press invested group and using only those data points whose range overlapped with the sunk cost group being compared. For example, in our 5xFR6 context, the 1 press invested group has data points that extend up to 47 presses remaining; however, the 5 presses invested group only extends to 43 presses remaining. Therefore, we take the regression of the full dataset for the 5 presses invested and compare it to a new regression of the 1 press invested group restrained to 1-43 presses remaining. **A.** Control condition (1 press invested) data points and linear fit in the 5xFR6 context. Dashed colored lines represent the highest press remaining that each sunk cost group extends. Thus, only the data points between the y-axis and the corresponding dashed line will be used in the adjusted control. **B.** Data points and regressions for each sunk cost group plotted against a new adjusted control regression calculated based on the 1 press invested points limited to the overlapping data range. |

**Table S1. Trials and rewards for each parameter.**

| \|  \| **2xFR6** \| **2xFR12** \| **5xFR6** \| **5xFR12** \| \| --- \| --- \| --- \| --- \| --- \| \| **Total Trials** \| 590 ± 120 \| 380 ± 60 \| 540 ± 80 \| 320 ± 100 \| \| **Completed Trials** \| 550 ± 130 \| 310 ± 60 \| 500 ± 90 \| 270 ± 100 \| \| **Reward Collected (µL)** \| 1700 ± 400 \| 1100 ± 200 \| 1900 ± 300 \| 1400 ± 300 \| |
| --- | --- | --- | --- | --- | --- | --- | --- | --- | --- | --- | --- | --- | --- | --- | --- | --- | --- | --- | --- | --- |
| Shows the average and standard deviation (n = 10 mice) of total number of trials, number of trials where reward was collected, and the total amount of reward collected. Fewer trials were done and less reward was collected in the FR12 conditions, which is likely due to the increased amount of time it takes to press the lever 12 times. |

**Legends for Movies**

**Movie S1.** An example of a mouse pressing a lever on the FR side with a small reward. The mouse must press 6 times to collect reward.

**Movie S2.** An example of a mouse pressing a lever on the PR side with a large reward. Three consecutive trials were presented. The mouse must first press 2, then 3, then 4 times to collect reward.
